# Monitored Anesthesia Care in Minimally Invasive Spine Surgery—A Retrospective Case Series Study

**DOI:** 10.3390/medicina60010043

**Published:** 2023-12-26

**Authors:** Hyo Jin Kim, Seongho Park, Yunhee Lim, Si Ra Bang

**Affiliations:** 1 Department of Anesthesiology and Pain Medicine, Chung-Ang University Gwangmyeong Hospital, Chung-Ang University College of Medicine, Gwangmyeong-si 14353, Republic of Korea; 2 Department of Anesthesiology and Pain Medicine, Inje University Sanggye Paik Hospital, Inje University College of Medicine, Seoul 01757, Republic of Korea

**Keywords:** dexmedetomidine, monitored anesthesia care, minimally invasive spine surgery

## Abstract

*Background and Objectives:* Minimally invasive spine surgery (MISS) under monitored anesthesia care (MAC) has emerged as a treatment modality for spinal radiculopathy. It is essential to secure the airway and guarantee spontaneous respiration without endotracheal intubation during MISS in a prone position. *Materials and Methods:* To evaluate the feasibility and safety of MAC with dexmedetomidine during MISS, we retrospectively reviewed clinical cases. A retrospective review of medical records was conducted between September 2015 and June 2016. A total of 17 patients undergoing MISS were included. Vital signs were analyzed every 15 min. The depth of sedation was assessed using the bispectral index (BIS) and the frequency of rescue sedatives. Adverse events during anesthesia, including bradycardia, hypotension, respiratory depression, postoperative nausea, and vomiting, were evaluated. *Results:* All cases were completed without the occurrence of airway-related complications. None of the patients needed conversion to general anesthesia. The median maintenance dosage of dexmedetomidine for adequate sedation was 0.40 (IQR 0.40–0.60) mcg/kg/hr with a median loading dose of 0.70 (IQR 0.67–0.82) mcg/kg. The mean BIS during the main procedure was 76.46 ± 10.75. Rescue sedatives were administered in four cases (23.6%) with a mean of 1.5 mg intravenous midazolam. After dexmedetomidine administration, hypotension and bradycardia developed in six (35.3%) and three (17.6%) of the seventeen patients, respectively. *Conclusions:* MAC using dexmedetomidine is a feasible anesthetic method for MISS in a prone position. Hypotension and bradycardia should be monitored carefully during dexmedetomidine administration.

## 1. Introduction

Degenerative spinal disease is on the rise as the aging population increases [1]. Accordingly, the number of spinal fusion surgeries in developed countries has steadily increased [2,3]. Minimally invasive spine surgery (MISS) has increased in popularity as an alternative to open surgery over the past two decades [4]. Recognized for its numerous advantages, including reduced blood loss, diminished postoperative pain, lower surgical site infection rates, and shorter hospital stays, MISS has become an attractive option for both patients and doctors [5,6,7].

Traditionally, spine surgery has conventionally been performed under general anesthesia [8]. However, with the emergence of enhanced recovery after surgery (ERAS) concept, a recent study demonstrated the endoscopic minimally invasive transforaminal lumbar interbody fusion technique performed without general anesthesia [5]. Monitored anesthesia care (MAC) consists of sedation, analgesia, and anxiolysis, distinguished from general anesthesia in that a patient’s spontaneous respiration and protective reflexes are maintained [9]. This approach allows for a lighter level of anesthesia compared to general anesthesia, potentially enhancing patient recovery speed and reducing hospitalization duration. This trend signifies a progression from the traditional use of general anesthesia in spine surgery towards lighter sedation methods like MAC. One of the recent sedatives used in MAC is dexmedetomidine (DEX). DEX is a lipophilic imidazole derivative and highly selective α-2 adrenergic receptor agonist that exerts sedative and analgesic effects with minimal respiratory depression [10]. Since spine surgery is performed in the prone position without endotracheal intubation, it is critical to secure the airway and maintain spontaneous respiration during surgery.

There has been an increased prevalence of outpatient MISS over the past two decades, and the growing challenges associated with limited resources and an aging population have been evident [11]. Compounding these challenges is the absence of a standardized anesthesia regimen for ERAS following spine surgery. In light of this gap, our study was undertaken to evaluate the feasibility and safety of employing MAC with dexmedetomidine during MISS, drawing insights from our initial clinical experiences.

## 2. Materials and Methods

This study was approved by the Institutional Review Board of the College of Medicine, Inje University Seoul Paik Hospital (IRB no. 2016-05-002-004). The requirement of informed consent was waived since we used deidentified administrative claimed data. We retrospectively reviewed the medical records of patients who underwent percutaneous endoscopic lumbar resection (PELD) and laminoplasty under monitored anesthesia care (MAC) at the Educational Medical Center from September 2015 to June 2016. Patients under the age of 20 were excluded.

The demographics and baseline characteristics, including age, sex, height, weight, body mass index (BMI), American Society of Anesthesiologists (ASA) classification, and past medical histories, were collected. To evaluate the hemodynamic stability, systolic blood pressure (SBP), diastolic blood pressure (DBP), heart rate (HR), and saturation of percutaneous oxygen (SpO2) were measured at intervals of 15 min from the beginning of the anesthesia. The depth of sedation was monitored using the bispectral index (BIS). The frequency of rescue sedatives was also recorded. Conversion to general anesthesia during MAC, hypotension, bradycardia, respiratory depression, nausea, and vomiting were identified.

At our institution, the standard MAC protocol for MISS was applied. In patients with moderate to severe anxiety, 1–2 mg of intramuscular midazolam was administered one hour before arriving in the operating room. Oxygen (3 L/min) was supplied via nasal prong and controlled at the discretion of the anesthesiologist in charge. The patient’s spontaneous respiration was monitored with capnography using a side stream capnometer mechanical ventilator (Dräger, Lübeck, Germany). After placement in a prone position on a pillow, the patient’s head was secured in a direction with which the patient felt comfortable. While draping the surgical field in a prone position, 0.7 mcg/kg DEX was loaded for ten minutes, followed by a maintenance dose of 0.4 mcg/kg/hr. Dose titration was aimed at a Richmond agitation sedation scale (RASS) level of −2 [12]. The target depth of sedation/analgesia was determined according to moderate sedation/analgesia, also known as conscious sedation, as defined by the ASA [13]. It refers to a state of reduced consciousness in which the patient purposefully responds to verbal commands alone or accompanied by light tactile stimulation. The inadequate level of sedation was defined when (1) the patient unexpectedly awakens, (2) is unable to cooperate with the surgical procedure due to agitation, or (3) fails to adhere to verbal commands of medical staff. If an adequate level of sedation was not achieved, 1 mg midazolam was additionally administered as directed by the anesthesiologist in charge. Then, 50 mcg of fentanyl was administered just before the skin incision as preemptive analgesia. Prior to the annulotome, 50 mcg of fentanyl was injected intravenously to alleviate the severe pain arising from the annulotome. Once the skin sutures began, the continuous infusion of DEX ceased. Hypotension was determined when there was more than a 30% decrement in baseline SBP or when the SBP was less than 90 mmHg. In the case of hypotension, 5 mg ephedrine or 50–100 mcg phenylephrine was administered intravenously. If hypotension persisted despite administering three or more boluses of ephedrine or phenylephrine, a continuous infusion of phenylephrine was started. Bradycardia was defined as more than a 30% decrease in HR compared to the baseline HR or when the HR was less than 45 beats/min. Bradycardia was treated with 0.25–0.5 mg atropine or 0.2 mg glycopyrrolate intravenously.

Conversion to general anesthesia was planned in the following cases: (1) moderate to severe intraoperative bleeding, (2) hypoxemia defined as SpO_2_ < 90% despite adequate oxygenation, (3) severe hemodynamic instability despite a continuous infusion of vasopressors and inotropes, (4) intractable arrhythmia, and (5) failed sedation resulting in the inability to proceed with the operation despite the repetitive administration of rescue sedatives and analgesics.

After surgery, the patients were transferred to the postanesthetic care unit (PACU). Postoperative nausea and vomiting (PONV) were treated with 0.3 mg ramosetron or 0.075 mg palonosetron intravenously. Patients with a modified Aldrete score of nine or greater were transferred to the general ward under confirmation by an anesthesiologist in charge.

Statistical analysis was performed using IBM SPSS Statistics for Windows software (version 20, IBM SPSS^®^ Software, Chigaco, IL, USA). In all analyses, *p* < 0.05 was judged to be a statistically significant value. For continuous variables, a Shapiro–Wilk test was performed to test normality. Depending on the distribution, continuous variables were analyzed with the Mann–Whitney U test and independent *t*-test. Continuous variables are expressed as the mean ± standard deviation (SD) or median and interquartile range (IQR). Categorical variables were analyzed using the chi-square test and described as frequency and percentage. The graphs were generated using the GraphPad Prism for windows software (version 10.1.2, GraphPad Software^®^, Boston, MA, USA).

## 3. Results

A total of 17 patients were included, and their medical records were reviewed retrospectively. The baseline characteristics are shown in Table 1. The median age of patients was 67 (IQR 63–80) years, including 12 males and 5 females; 29.4% (*n* = 5) of patients were classified as American Society of Anesthesiologists (ASA) classification 1, 64.7% (*n* = 11) were classified into ASA classification 2, and the remaining 5.9% (*n* = 1) were classified into ASA classification 3. The previous medical history of patients included hypertension (35.3%, *n* = 6), diabetes (17.6%, *n* = 3), arrhythmia (11.8%, *n* = 2), coronary artery disease (5.9%, *n* = 1), asthma (5.9%, *n* = 1), and liver disease (5.9%, *n* = 1). Twelve patients were diagnosed with spinal stenosis (70.6%), three with a herniation of an intervertebral disc (17.6%), one with facet joint syndrome (5.9%), and one with a compression fracture (5.9%).

The operation and anesthesia details are demonstrated in Table 2. Fourteen patients underwent dome laminoplasty (82.4%), whereas three patients underwent percutaneous endoscopic lumbar discectomy (17.6%). The median operation time was 140 (IQR 130–155) minutes, and the median anesthesia time was 185 (IQR 170–200) minutes. The median maintenance dosage of dexmedetomidine for adequate sedation was 0.40 (IQR 0.40–0.60) mcg/kg/hr with a median loading dose of 0.70 (IQR 0.67–0.82) mcg/kg. The median total dose of dexmedetomidine was 1.93 (IQR 1.72–2.37) mcg/kg.

**Figure 1:**
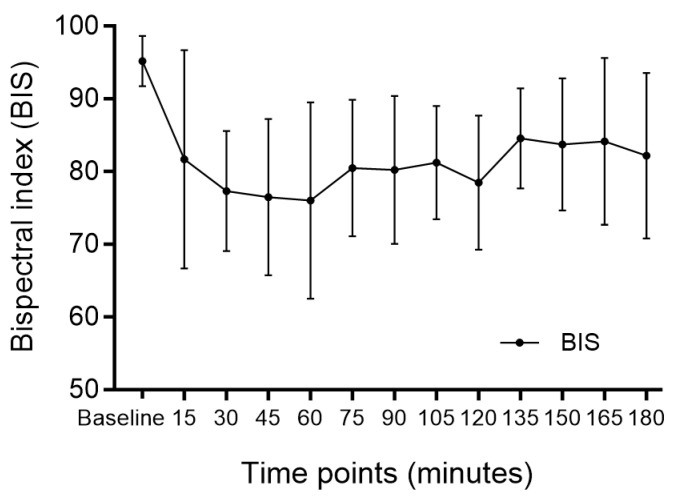
Depth of sedation assessed using the bispectral index. BIS; bispectral index.

**Figure 2:**
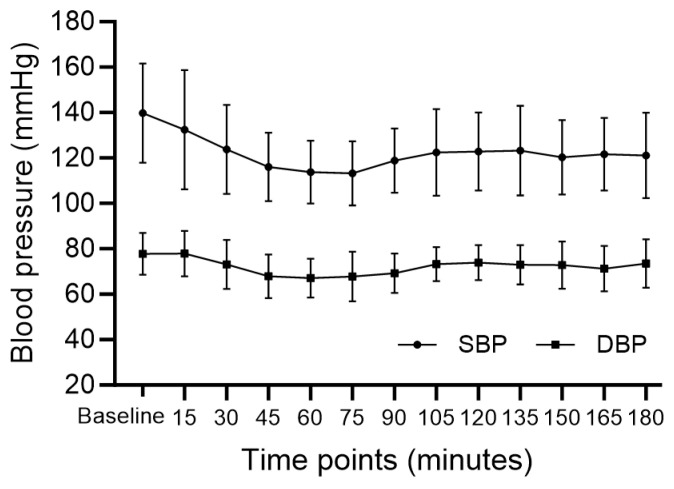
Changes in blood pressure. SBP; systolic blood pressure, DBP; diastolic blood pressure.

**Figure 3:**
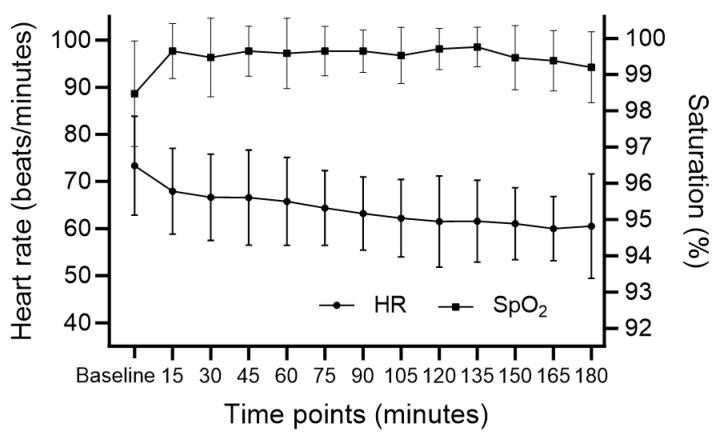
Changes in heart rate and percutaneous oxygen saturation. HR; heart rate, SpO_2_; percutaneous oxygen saturation.

The changes in BIS are shown in Figure 1. The mean BIS during the main procedure was 76.46 ± 10.75. Rescue sedatives were administered in four cases (23.6%) with a mean of 1.5 mg intravenous midazolam. Changes in blood pressure and HR during surgery are presented in Figure 2 and Figure 3, respectively. After DEX administration, hypotension and bradycardia developed in six (35.3%) and three (17.6%) of the seventeen patients, respectively (Table 3). None of the patients needed conversion to general anesthesia. All cases were completed without the occurrence of airway-related adverse events.

## 4. Discussion

In this case series, anesthesia was maintained successfully without converting to general anesthesia and without desaturation. However, during the administration of DEX, there were treatable hypotensive and bradycardia events. Being female, obesity, and underlying hypertension have been reported as risk factors for hemodynamic instability associated with dexmedetomidine [14]. In the present study, all patients except for one belonged to ASA PS classification 1 or 2, and six patients had underlying hypertension. An essential aspect of ensuring patient safety in MAC is the meticulous monitoring of the level of sedation. The level of sedation was confirmed through the BIS during surgery and was maintained at an appropriate level for the surgical procedure.

MISS has gained prominence due to its associated benefits, including reduced blood loss, decreased postoperative pain, lower infection rates, and shorter hospital stays [5,6,7]. Traditionally, spine surgeries were conducted with patients in the prone position under general anesthesia. However, as the concept of ERAS emerged, there has been a paradigm shift toward exploring alternative anesthesia approaches for MISS. Historically, the conventional approach involved general anesthesia for spine surgeries, ensuring a deep level of sedation to facilitate the procedure. However, the evolution of anesthesia in MISS has seen a transition from deep sedation to more targeted approaches. Local anesthesia [15], epidural anesthesia [16], or MAC [17] have emerged as viable alternatives, offering advantages such as faster recovery and cost-effectiveness.

Currently, MAC is the preferred method of outpatient anesthesia over general or regional anesthesia because of its rapid recovery and low cost [18,19]. After careful monitoring of the level of consciousness and hemodynamic variables, sedatives and analgesics should be infused. These reagents can be selected based on the type of procedure. We believe that MISS is an adequate procedure for MAC because it is minimally invasive and enables an early recovery and discharge feasible for ERAS. Cooperative conscious sedation allows for self-reporting of potential nerve damage and ensures spontaneous respiration. However, the prone position might cause airway obstruction, and thus, MAC may not be the anesthesiologist’s first choice during MISS [20].

Choosing an appropriate anesthetic agent is pivotal for ensuring patient safety, comfort, and optimal surgical conditions. DEX, a highly selective α-2 adrenergic receptor agonist, has gained prominence as a major sedative agent in MAC due to its favorable profile. The choice of DEX as a major sedative in our study was driven by its efficacy in protecting the airway during surgery performed in the prone position without endotracheal intubation. It is known that DEX is an effective primary sedative for patients undergoing MAC for various surgical procedures, providing better patient satisfaction, fewer opioid requirements, and less respiratory depression than the traditional combination of benzodiazepine and opioids [21]. A recent retrospective study in elderly patients who underwent orthopedic surgery showed that intraoperative DEX sedation reduces postoperative agitation compared to propofol sedation and does not induce postoperative delirium [22]. Postoperative delirium can be a major complication of surgery, especially in elderly patients. Therefore, DEX may be a better option for anesthesia for those who have concerns about postoperative delirium. There are various surgical procedures that require anesthesia. DEX is suitable for MISS for its analgesic-sparing effect and may potentially reduce the risk of developing respiratory depression that is anticipated in the prone position.

Multimodal analgesia and ERAS pathways of care have become standard methods to reduce opioid use and related side effects and improve postoperative outcomes in orthopedic surgery [23,24]. In a recent study by Soffin et al., DEX was shown to be a good candidate for opioid-free anesthesia for MISS [25]. We believe this study offers case-based evidence that may help solve some problems in anesthetic care.

In this study, the administration of atropine in the range of 0.25–0.5 mg was employed as a part of our protocol for managing bradycardia during the surgeries. In an elderly patient with asymptomatic bradycardia without hypotension or low body weight, administration of 0.25 mg of atropine was considered due to the risk of severe tachycardia. Research findings have reported that a low dose of atropine can induce paradoxical bradycardia by affecting central muscarinic receptors, potentially influencing sinoatrial node activity or increasing vagal nerve activity [26,27]. While our initial goal was to manage asymptomatic bradycardia in elderly patients exhibiting little to no hypotension using a 0.25 mg dose of atropine, we acknowledge the necessity to refine the atropine administration protocol for the treatment of bradycardia.

This study has some limitations. First, the number of patients included in this case study is too small. A larger clinical study is needed to confirm the efficacy of MAC in MISS. Second, because of the retrospective design of the study, we could not include data on patient satisfaction. Lastly, a noteworthy limitation of this study lies in its focus on only two specific MISS techniques, namely dome laminoplasty and PELD. Consequently, the findings may not be readily generalizable to other MISS techniques, warranting caution in broader applications. To overcome this constraint, future investigations should endeavor to include a broader spectrum of MISS scenarios for a more comprehensive understanding of the efficacy and safety of MAC with DEX. Subsequent studies should also undertake a comparative analysis between patients treated with DEX and a carefully chosen control group to yield more robust and generalizable insights. Nevertheless, our present study underscores the evolving landscape of anesthesia in MISS. The choice of MAC using DEX in this study is supported by a thorough consideration of patient characteristics, the level of sedation monitoring, and the advantages offered by DEX in terms of airway protection and analgesic effects. As we navigate the complexities of anesthesia in spine surgery, further research, and larger-scale studies are warranted to solidify the evidence base for optimal anesthetic practices in the realm of MISS.

## 5. Conclusions

MAC using DEX is a feasible anesthetic method for MISS in a prone position. It is recommended that hypotension and bradycardia should be monitored carefully during DEX administration, especially during the loading period.

## Figures and Tables

**Table 1:** Baseline characteristics.

	Value
Age, years	67 [63–80]
Sex	
Male	12 (70.6%)
Female	5 (29.4%)
Height, cm	161.9 [158.3–168.2]
Weight, kg	66.6 [58.0–71.8]
BMI, kg/m^2^	25.1 [22.1–26.5]
ASA PS	
1	5 (29.4%)
2	11 (64.7%)
3	1 (5.9%)
Previous medical history	
Hypertension	6 (35.3%)
Diabetes	3 (17.6%)
Arrhythmia	2 (11.8%)
Coronary artery disease	1 (5.9%)
Asthma	1 (5.9%)
Liver disease	1 (5.9%)
Diagnosis	
Spinal stenosis	12 (70.6%)
HIVD	3 (17.6%)
Facet joint syndrome	1 (5.9%)
Compression fracture	1 (5.9%)

Values are presented as numbers (%) and medians [interquartile range]. BMI; body mass index, ASA PS; American Society of Anesthesiologists physical status, HIVD; herniation of inter-vertebral disc.

**Table 2:** Profiles of anesthesia and surgery.

	Value
No. of operated spine levels	
1	14 (82.6%)
2	2 (11.8%)
3	1 (5.9%)
Type of surgery	
Dome laminoplasty	14 (82.4%)
PELD	3 (17.6%)
Duration of surgery, mins	140 [130–155]
Duration of anesthesia, mins	185 [170–200]
Recovery time, mins	62.0 [47.0–72.0]
Premedication	
Midazolam	7 (41.2%)
Glycopyrrolate	2 (11.8%)
None	8 (47.0%)
Dexmedetomidine	
Loading dose, mcg/kg	0.70 [0.67–0.82]
Maintenance dose, mcg/kg/hr	0.40 [0.40–0.60]
Total infused dose, mcg/kg	1.93 [1.72–2.37]
Need for rescue sedative	4 (23.6%)

Values are presented as numbers (%) and medians [interquartile range]. PELD; percutaneous endoscopic lumbar discectomy.

**Table 3:** Adverse events.

	Value
Hypotension (SBP < 90 mmHg)	6 (35.3%)
Bradycardia (HR < 45 bpm)	3 (17.6%)
Need for continuous infusion of phenylephrine	3 (17.6%)
Conversion to general anesthesia	0 (0%)
Postoperative nausea and vomiting	3 (17.6%)

Values are presented as numbers (%). SBP; systolic blood pressure, HR; heart rate.

## Data Availability

No new data were created or analyzed in this study.
